# The impact of enhanced cleaning on bacterial contamination of the hospital environmental surfaces: a clinical trial in critical care unit in an Egyptian hospital

**DOI:** 10.1186/s13756-024-01489-z

**Published:** 2024-11-19

**Authors:** Nermine Mahmoud Hassan Hamed, Osama Ahmed Deif, Aleya Hanafy El-Zoka, Magda Mohamed Abdel-Atty, Mohamed Fakhry Hussein

**Affiliations:** 1 Alexandria University Hospitals, Alexandria University, Alexandria, Egypt; 2 Neurosurgery Department, Faculty of Medicine, Alexandria University, Alexandria, Egypt; 3 Environmental Health, High Institute of Public Health, Alexandria University, Alexandria, Egypt; 4 Environmental Chemistry and Biology, High Institute of Public Health, Alexandria University, Alexandria, Egypt

**Keywords:** Bacterial contamination, Enhanced cleaning, Healthcare acquired infection, Infection prevention and control, Healthcare facilities, Nosocomial infections

## Abstract

**Background:**

Contaminated environmental surfaces play an important role in the transmission of pathogens that cause healthcare acquired infection (HAI). The present study aimed to assess the effect of enhanced cleaning techniques on bacterial contamination in high-touch areas compared to routine cleaning at the intensive care units (ICU) of the neurosurgery department of Alexandria Main University Hospital, Egypt.

**Methods:**

The assessment of the knowledge and practices of healthcare cleaning workers and nurses was conducted through a questionnaire and an observational checklist. An educational program about enhanced cleaning was carried out for healthcare cleaning workers and nurses in one room of the ICU unit. Environmental surface swabs were taken from the two rooms of the ICU before and after cleaning (room A and room B). Room A was selected to apply the enhanced cleaning, and room B was selected for routine cleaning.

**Results:**

A significant decrease in bacterial counts in the high-touch areas around the patients after the application of enhanced cleaning compared to routine cleaning (*p* < 0.001) was observed. Gram-negative bacteria isolated from high-touch areas accounted for 45.6% of the samples collected before enhanced cleaning, and they became 16.3% after enhanced cleaning (*p* < 0.001), while they accounted for 40% after routine cleaning. The enhanced cleaning intervention in Room A resulted in a significant reduction in total infections, decreasing from 18 cases in the six months prior to the intervention to 11 cases in the six months following its implementation. (*p* < 0.05).

**Conclusion:**

The effect of enhanced cleaning was evident in decreasing bacterial counts in the high-touch areas around the patient and consequently in the records of the HAI rate inside the ICU.

**Clinical trial registration number:**

PACTR202402531001186, date: 15 February 2024, ‘retrospectively registered’.

**Supplementary Information:**

The online version contains supplementary material available at 10.1186/s13756-024-01489-z.

## Background

Hospitalization, while intended to improve health, can also lead to infections. Healthcare acquired infection (HAI), those acquired during a hospital stay, are the most frequent complication of healthcare. In the United States alone, an estimated 4.5 million HAI occur annually, resulting in roughly 147 thousand deaths [1]. In low-income and middle-income countries (LMICs), up to 25% of inpatients may be at risk for HAI. Several factors contribute to the elevated rates of HAI in these countries. These include inadequate infrastructure, insufficient surveillance, a shortage of trained healthcare workers and inadequate infection control training, limited or unsuitable equipment, work overload, high staff turnover, and inadequate disinfection practices [2, 3].

One critical factor in HAI transmission is contamination of the hospital environment itself. Studies suggest that contaminated surfaces play a role in 25 to 32.7% of HAI within intensive care units (ICU) [4, 5].

Gram-negative bacteria are a particular concern. These organisms can be shed from infected patients and linger on surfaces despite routine cleaning and disinfection, making them difficult to eradicate [6]. Escherichia coli and Pseudomonas aeruginosa are particularly common culprits in HAI, alongside Klebsiella, Proteus, Acinetobacter, and Enterobacteriaceae [7]. However, the threat extends beyond these. Pathogens such as Staphylococcus aureus, Streptococcus, Clostridium difficile, and even Candida albicans can also contaminate the environment and cause HAI [7]. The rise of antibiotic resistance also underscores the urgent need for effective prevention and control strategies [6–8].

The research by Russotto et al. (2015) shines a light on a major challenge: keeping surfaces clean in ICUs. They found that frequently touched surfaces and equipment around patients’ beds are particularly prone to bacterial contamination. This makes sense, considering the ICU environment. The patient’s surroundings are often crowded with equipment for monitoring and supporting vital functions, like monitors, ventilators, and even extracorporeal life support machines. Cleaning such a complex environment effectively requires specialized techniques [9].

Specialized cleaning protocols as enhanced cleaning in ICU focus on frequently touched surfaces and involves proper cleaning and disinfection techniques using appropriate solutions [10, 11]. Scientists have proposed using the aerobic colony counts, to gauge the effectiveness of cleaning in these high-risk areas. The ideal range is between 2.5 and 5 colony-forming units (CFU) per square centimeter on frequently touched surfaces [12]. Sodium hypochlorite solution is widely used disinfectant because it is inexpensive and effective broad-spectrum germicidal solution [13].

There is evidence that enhanced cleaning aids in the control and prevention of HAI. Many studies have found that enhanced environmental cleaning, which involves more frequent and detailed cleaning, can significantly reduce the spread of bacteria and consequently decrease HAI. They documented that any shortage in the process is associated with an increase in the incidence of HAI [11, 14, 15].

A multi-factorial approach also is essential for reducing HAI rates. Enhanced cleaning is a crucial component, but it needs other supportive means. Training healthcare workers on infection prevention and control (IPC) practices, including proper hand hygiene, is paramount. Regular auditing of compliance with these practices helps identify and address gaps. Moreover, ensuring strict adherence to hand hygiene guidelines is vital, as it serves as a cornerstone of infection prevention. By combining these elements, a more comprehensive and effective strategy can be implemented to combat the spread of infections [16–18].

Salem and Youssef conducted an Egyptian study at Cairo University Hospitals in 2017. They reported high rates of HAI in the neonatal ICU due to a lack of time to implement IPC standards, limited opportunities for IPC training, inadequate environmental cleaning, and work overload [19]. So, the implementation of strict IPC procedures, including proper environmental disinfection, is mandatory to reduce HAI and improve the quality of hospital care.

## Methodology

### Aim of the study

The aim of this study was to assess the current status of the routine cleaning of the environmental surfaces at the intensive care unit of the Neurosurgery Department in Alexandria Main University Hospital, Egypt, and to evaluate the impact of enhanced cleaning of the environmental surfaces on bacterial contamination in the same setting.

### Study design and setting

A pre- and post-interventional design was conducted in this study at the intensive care unit of the neurosurgery department at Alexandria University Main Hospital, Egypt (room A with eight beds and room B with six beds). The two rooms were having the routine cleaning once every day in the morning at 10 a.m. The present study was conducted between September 2020 and February 2021.

The ICU in this study was receiving about 25 patients per month. It is primarily serves adult patients who have undergone neurosurgery. Common cases include intracranial hemorrhage, brain tumors, and brain abscesses. Some patients with brain stem compression may experience weak gag reflexes, leading to aspiration pneumonia or the need for mechanical ventilation. Central lines are frequently inserted in these patients. Common ICU-acquired infections include ventilator-associated pneumonia, central line infections, and surgical site infections. While the average length of stay in the ICU is typically 3 to 7 days, which may not be enough to cause the ICU-acquired infections, some patients may require prolonged care for up to 30 days or more. The American Society of Anesthesiologists (ASA) score for patients in this ICU generally ranges from 2 to 5, indicating varying degrees of health status and surgical risk. So, it is of great importance to apply an effective environmental cleaning technique.

### Target population and materials

All healthcare cleaning workers and nurses were recruited for the study. Ten workers and eight nurses are responsible for each ICU room. Samples were taken from ICU environmental surfaces: from the high-touch areas around each bed (side tables, suction machines, medical devices, headboard of the bed, side rails, footboard, light switch, etc.), doorknobs, and nurse counters. The materials used in the hospital-built environment, particularly in the ICU, while durable, such as stainless steel, marble, and ceramic, are not entirely new, and some have cracks or broken that could harbor bacteria if not properly cleaned and disinfected. Sinks are located outside the ICU. These factors emphasize the importance of rigorous cleaning and disinfection practices to prevent the spread of infections. Hospital records were used to extract the HAI rates in the ICU units before and after the intervention.

### Sampling design

For the healthcare cleaning and disinfection team, half of the healthcare cleaning workers and nurses (those who were responsible for room A cleaning) were included in the application of the enhanced cleaning technique of the intervention phase (eighteen persons), and the other half, who were responsible for room B cleaning, did not receive any new instructions about cleaning (except after the end of the research for ethical consideration). For environmental sampling, 736 environmental surface swabs were taken (416 samples from room A and 320 from room B) (Table 1) through convenient sample techniques. Three samples from the area around each bed before and after cleaning were taken. Besides, one swab from the nursing counter and another one from the doorknob were taken. This process was repeated twice per week for 4 weeks.

**Table 1:** Distribution of samples taken from ICU rooms

ICU (2 rooms)	Number of swabs location*number of swabs*2 (before and after cleaning)	Repetition of the sampling	Total number of swabs for each room	Total number of swabs
***Room A***		**8 times** (Twice per week and repeated in 4 weeks)	416	**736**
Area around each bed (8 beds)	8*3*2 = 48			
Nursing counter (one)	1*1*2 = 2			
Doorknob (one)	1*1*2 = 2			
***Room B***			320	
Area around each bed (6 beds)	6*3*2 = 36			
Nursing counter (one)	1*1*2 = 2			
Doorknob (one)	1*1*2 = 2			

### Data collection method and tools

#### Predesigned interview questionnaire (supplementary file I)

The questionnaire consisted of two parts. The first part was concerned with the socio-demographic data (sex, age, education, occupation, marital status). The second part assessed the participants’ knowledge of the significance of hand hygiene and the appropriate times for hand-washing. Additionally, we assessed their understanding of standard and transmission-based precautions. Also, we asked about cleaning technique in details. The questionnaire includes items about frequency and schedules of cleaning, cleaning methods, direction of cleaning, disinfectant used and its concentration, materials and equipment used (including cleaning cloths, dust mops, disinfectants, and scrubbers), how to clean the equipment after finishing the cleaning process, and the proper technique to clean human spills. Previous training about cleaning and disinfection, the presence of supervision during cleaning, and the purpose of the cleaning and disinfection of healthcare facilities were also asked.

The questionnaire was obtained from previously validated research questionnaires [20–22]. Two professors (in public health and infection prevention and control) revised the questionnaire to check the content validity and recommended a few modifications, which included replacing and adjusting some questions. The English questionnaire was translated into Arabic by two separate native speakers who are specialists in public health.

A ‘1’ point was given for the right response, while a ‘0’ point was given for the wrong response. For the multiple correct answer questions, complete correct answer received ‘2’ points, incomplete correct answer took‘1’ point and incorrect answer received ‘0’ point. After adding together all of the scores, a percentage between 0 and 100% was calculated. Three categories have been established for the knowledge and practice level assessment: low (0 ≤ 50%), fair (50 ≤ 70%), and good (70–100%).

#### Observational checklist

An observational checklist obtained from the Centers for Disease Control and Prevention (CDC) [23] (Supplementary File II) was used to assess the practice of the healthcare cleaning workers and nurses and to evaluate the level of cleaning visually. There is a list of the items that are present in the patient’s room that are considered high-touch surfaces. The checklist was based on categorizing the objects by three parameters: cleaned, not cleaned, or not present in the room. The rule of this categorization is whether the surface has dust or spills on it. This assessment was done visually immediately after the cleaning process. The visually clean item took ‘1’ point and the unclean one received ‘0’ point and the percentage of cleaned and dirty items was calculated.

#### Data collected from the records

The rates of HAI occurring in the ICU of the neurosurgery department 6 months before and after the application of the enhanced cleaning intervention were obtained from the IPC records.

#### Environmental samples

Swabs from environmental surfaces were collected using a steel template (25 cm^2^ sample area) as guided by the CDC [24]. Swabs were taken from the high-touch areas in the two intensive care rooms A and B (3 swabs from the high-touch areas around each bed, 1 swab from the counter, and 1 swab from the doorknob) before and after cleaning and repeated two times per week for four weeks. The intervention was applied in room A, as it contains a higher number of beds than room B (8 beds vs. 6 beds). The samples from the two rooms were matched to be taken at the same time on the same days.

The collected swabs were immediately transported to the lab and cultured on nutrient-agar plate media. The samples were incubated at 37° C for 48 h, and the total aerobic colony count (TACC) was counted using a colony counter. The microbiological cut-off level for environmental surface contamination was set at 5 CFU per cm^2^. Colonies from the cultured plates were taken randomly to carry out Gram staining to detect Gram-negative and Gram-positive bacteria [12, 25].

### Pilot study

A pilot study was carried out before the implementation of the actual study to assess the status of the routine cleaning of the environmental surfaces in the high-touch areas in the two intensive care rooms A and B (3 swabs from high-touch areas around each bed, 1 swab from the counter, and 1 swab from doorknob), for a total of 184 swabs. Also, it was conducted to determine the practicability of the tool used (environmental swabs) and identify obstacles that could be faced during the implementation of the study. It helped in identifying the preferred time and locations to collect the samples. It entailed taking samples from high-touch areas in the study setting before and after routine cleaning. The geometric means of all the samples before and after cleaning were above the allowable limit in all samples (above 5 CFU/cm^2^), indicating the urgent need for intervention.

### Educational program about enhanced cleaning

When observing the routine cleaning and after data extraction from the questionnaire we found inconsistent cleaning procedures, including the absence of regular detergent use prior to disinfection, improper disinfectant concentration and handling, the directions of cleaning were haphazard, the cleaning mop did not exchange frequently, improper cleaning of the equipment after finishing the cleaning process, and absence of supervision during cleaning (routine cleaning).

To improve cleaning procedures in the ICU, we developed an educational program for healthcare cleaning workers and nurses responsible for cleaning and disinfecting Room A. The program consisted of three interactive sessions held within a month, each lasting 30–45 min. The training used audio-visual presentations and group discussions to emphasize the importance of enhanced cleaning and its key components. Enhanced cleaning consisted of a two-step process to effectively clean and disinfect surfaces and equipment. Initially, surfaces were cleaned with detergent to remove visible dirt and debris, creating an optimal environment for subsequent disinfection. The second step involved applying a suitable disinfectant at the correct concentration for the required contact time to eliminate microorganisms. The program educated the participants about the enhanced cleaning by covering the following points [22, 26]:

- **Hand hygiene**: The educational program emphasized the critical role of hand hygiene in preventing the spread of infections. Participants were instructed on the proper technique for hand washing, including using soap and water for at least 20 s, drying hands thoroughly, and using hand sanitizer when soap and water are not available. Key moments for hand hygiene were highlighted, such as before and after patient contact, before and after a clean procedure, and after using the bathroom. Additionally, the program addressed the importance of wearing gloves and disposing of them properly to prevent cross-contamination.
- **Standard and transmission-based precautions to minimize the risk of infection transmission.** Participants were instructed on the importance of wearing appropriate personal protective equipment (PPE), such as gloves, gowns, masks, and eye protection, depending on the situation. The program also covered the proper handling and disposal of contaminated materials, including soiled linens, medical waste, and bodily fluids. Additionally, participants were educated on respiratory hygiene practices, such as covering coughs and sneezes with a tissue or elbow, and maintaining a safe distance from others.
- **Dedicated Supplies**: We trained staff on the importance of using cleaning supplies designated specifically for the ICU and not shared with other areas. This included fresh mops and buckets with fresh cleaning solution for each cleaning session.
- **Proper Cleaning Techniques**: The program emphasized the importance of regularly changing cleaning cloths during disinfection and never reusing the same cloth in disinfectant solutions (avoiding “double-dipping”).
- **Cleaning Frequency**: Staff learned about the recommended cleaning schedule: disinfecting high-touch surfaces twice daily at a minimum, and more often as needed. Additionally, they were trained to change cleaning cloths between cleaning different patient zones within the room.
- **Disinfectant Preparation and Use**: The training covered the proper preparation of chlorine-based disinfectants, aiming for a concentration of 500–5000 parts per million (ppm) of free chlorine (which can be achieved by diluting 5% chlorine bleach at a ratio of 1:100 or 1:10). Staff also learned about the crucial “contact time” - ensuring the disinfectant remains wet on surfaces for at least 10 min to be sure of killing or inactivating serious microorganisms such as Mycobacterium tuberculosis, hepatitis B virus, or human immunodeficiency virus, especially in areas with high risk of infection like the ICU particularly in limited resources facilities.
- **Blood and Body Fluid Spills**: The program covered the proper response to blood and body fluid spills. This included immediate removal of spills using a special disinfectant designed for such situations (intermediate-level disinfectant). We also stressed avoiding the use of combination detergent-disinfectant products for spills.

#### Evaluation of the interventional program

To assess the effectiveness of the training program, environmental samples from high-touch areas in the ICU room before and after the intervention were collected. These samples were analyzed to measure TACC and stained by Gram staining to calculate the percentage of Gram-positive and Gram-negative colonies.

### Ethical considerations

The researcher sought the approval of the Ethics Committee of the High Institute of Public Health at Alexandria University, Egypt, for conducting the research. The researcher complied with the International Guidelines for Research Ethics. Informed consent was obtained from all study participants after an explanation of the purpose and benefits of the research. Anonymity and confidentiality were assured and maintained. The non-intervention group was educated after the end of the study. The clinical trial registration number is PACTR202402531001186.

### Statistical analysis

All the data collected from the questionnaire, records, and microbiological samples were entered into an Excel sheet. The collected data was subjected to statistical analysis using the Statistical Package for Social Science (SPSS) software package version 20.0. The median and interquartile range (IQR) were calculated for the knowledge score. Quantitative data on the bacterial counts were described using a geometric mean and log standard deviation (GM ± Log_10_SD). Simple frequency distribution tables along with GM ± Log_10_SD were used as descriptive analyses for bacterial counts. A cross-tabulation to compare the two groups of the study (Room A with enhanced technique and room B with routine technique) was used. The chi-squared test was used for calculating significant differences between the groups whenever possible. The student t-test was used to determine if there was a significant difference between the geometric means of the two groups regarding the quantitative data with a normal distribution. The Mann Whitney test (U) was used to test the statistical significance difference between two groups of continuous data with a non-normally distribution. The McNemar test is used to compare the proportions of paired categorical data, particularly when the outcomes are dichotomous, such as before and after intervention. So, The McNemar test was used in this study to compare infection rates before and after an enhanced cleaning intervention in each room. The significance of the obtained results was judged at the 5% level with a t-test and a chi-squared test.

## Results

There are ten workers and eight nurses in each room. According to the socio-demographic data of the workers (20 persons for both rooms, 10 for each room), five persons (25.0%) were between 18 and 30 years old, and fifteen persons were more than 30 years old. Half of them were males, 14 workers didn’t finish high school (70.0%), four workers (20.0%) finished high school, and two (10.0%) had university education. Besides, all of them were married. The mean age of nurses was 35 years. All of them were females and married. For each ICU room, two nurses had a higher than secondary school educational degree, and six had a secondary education or less. There was no significant difference between healthcare cleaning workers and nurses in both rooms regarding socio-demographic data (Supplementary File III: Table 1).

The assessment of knowledge and practices for the healthcare cleaning workers and nurses was carried out via an interview questionnaire to assess their awareness about hand hygiene, standard and transmission-based precautions, and cleaning status in the ICU. Questionnaire findings revealed a knowledge deficit regarding the link between proper hand hygiene and reduced HAI, with only 27.8% of participants demonstrating adequate understanding. However, over two-thirds exhibited acceptable knowledge levels of standard and transmission-based precautions. A shortage in information about proper cleaning procedures was also evident, potentially affecting their cleaning efficiency. According to the data collected from the healthcare cleaning workers and nurses, they were unaware of the effect of cleaning and disinfection on decreasing infections in the ICU (83.3%). Half of them (50.0%) were not following the proper technique of the cleaning procedure instructed by the CDC. About two-thirds of the healthcare cleaning workers and nurses (66.7%) answered wrongly about the proper liquids that should be used for ICU cleaning, and half of them (50.0%) had incorrect answers for proper disinfection fluid. The vast majority of the participants had wrong answers about the proper technique for dealing with spills (88.9%) (Supplementary File III: Table 2). Before the educational program, it was found that the proportion of participants with a good knowledge level was 5.6%, and more than half (58.3%) had poor knowledge. There was no statistical difference between the intervention and non-intervention groups regarding knowledge level (*p* > 0.05).

**Table 2:** Scores and levels of knowledge of the healthcare cleaning workers and nurses before the educational program

	Total participants *n* (%) (*n* = 36)	Participants under intervention *n* (%) (*n* = 18)	Participants with no intervention *n* (%) (*n* = 18)	Test of significance (*p*-value)
Score median (IQR)	51 (40–60)	52 (42–62)	53 (44–64)	U=-0.727 *p* = 0.465
Knowledge				
• Poor • Fair • Good	21 (58.3) 13 (36.1) 2 (5.6)	11 (61.1) 6 (33.3) 1 (5.6)	10 (55.5) 7 (38.9) 1 (5.6)	χ^2^ = 0.12 *p* = 0.939

IQR: Interquartile range U: Mann Whitney test *p* = *p*-value

### Observational checklist for the cleanliness of the high-touched objects

**Fig. 1:**
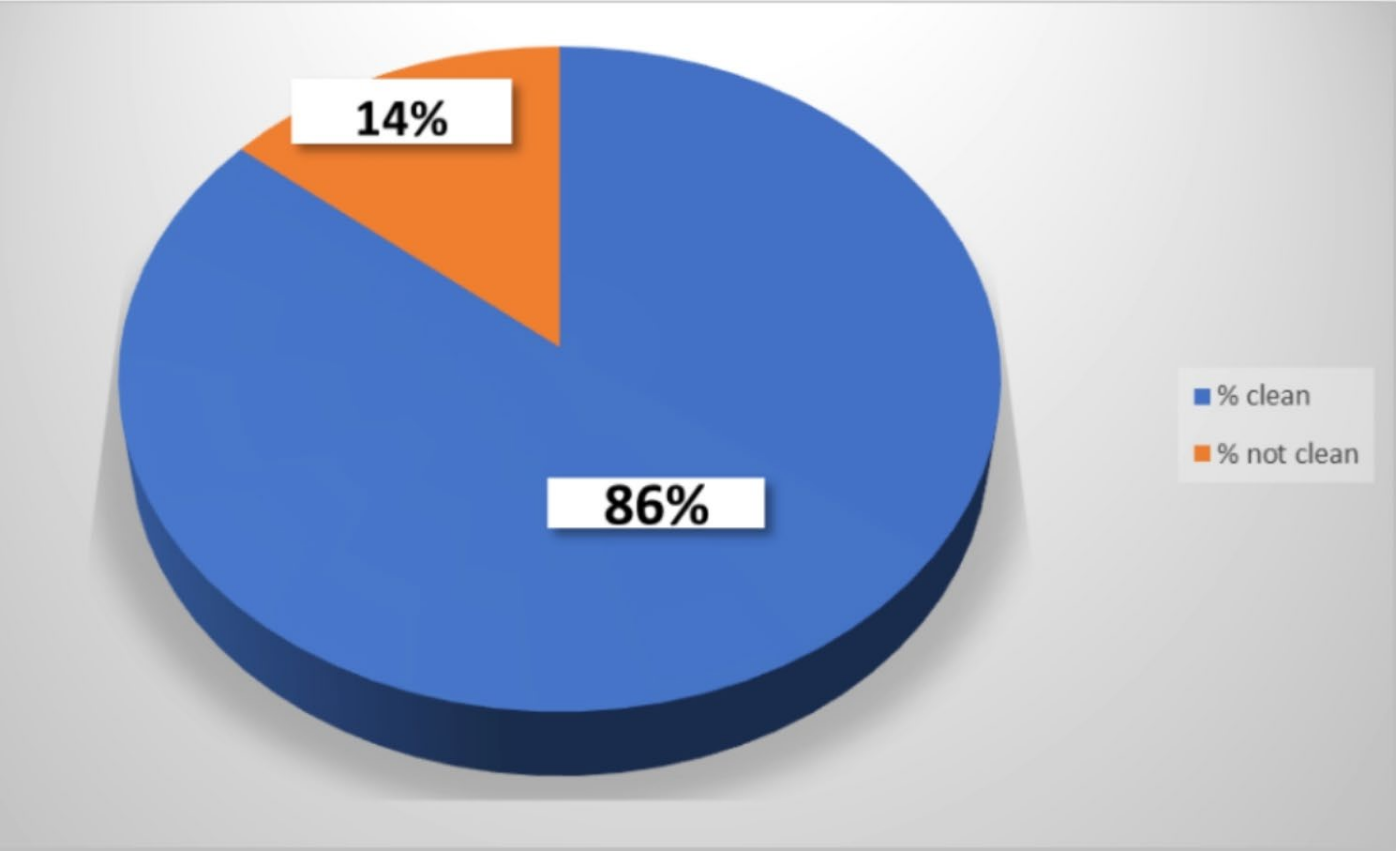
Assessment of the cleanliness of the high-touched objects in the ICU

According to the CDC observational checklist [20], a visual inspection revealed that 86% of the observed objects appeared clean. Any item that had any dirt or spills was considered not clean. Only 14% of the items checked were considered not clean (some of the tray tables and some of the ventilator screens) (Fig. 1).

Since a visual observation of the cleanliness of the environmental surfaces was insufficient to determine whether they were disinfected or not, particularly in the ICU, we collected environmental samples to go through the real situation of the cleaning process. Table 3 shows that the geometric means of the TACC of the samples collected from the areas around the beds, counters, and doorknobs in each room before applying any cleaning were similar in room A and room B, with no significant difference (*p* > 0.05) except for counters.

After cleaning, both rooms had significantly lower bacterial counts than before cleaning (*p* < 0.001), but only after the enhanced cleaning, the geometric mean of TACC became less than the upper permissible level (5 CFU/cm^2^). The percent reduction after the enhanced cleaning for high-touch areas around the beds was 91.4% in room A, while it was only 47% after routine cleaning in room B. Similar results were reported for the nursing counters and doorknobs, where the reduction in bacterial counts was below the recommended level only after the enhanced cleaning. The reduction in the geometric means of the TACC in the samples in room A was significantly greater than that of room B after cleaning (*p* < 0.05) in all areas examined.

**Table 3:** The geometric means of total aerobic colony count from high-touch areas

	Total Aerobic Colony Count CFU/cm^2^			t	*p*
	Before cleaning	After cleaning	% reduction		
	GM ± Log_10_SD	GM ± Log_10_SD			
**Samples from high touch areas around beds**					
**Room B** With routine cleaning	24.82 ± 0.43	13.15 ± 0.31	47.0	43.327^*^	< 0.001^*^
**Room A** With enhanced cleaning	25.54 ± 0.48	2.18 ± 0.00	91.5	105.012^*^	< 0.001^*^
**t(p)**	1.94 (0.125)	62.21^*^ (< 0.001)			
**Samples from counters**					
**Room B** (With routine cleaning)	24.85 ± 0.05	12.11 ± 0.19	51.2	27.835^*^	< 0.001^*^
**Room A** (With enhanced cleaning)	29.31 ± 0.33	3.02 ± 0.14	89.6	29.023^*^	< 0.001^*^
**t(p)**	5.28* (< 0.001*)	16.41* (< 0.001)			
**Samples from doorknobs**					
**Room B** (With routine cleaning)	17.71 ± 0.11	7.64 ± 0.29	56.86	11.929^*^	< 0.001^*^
**Room A** (With enhanced cleaning)	16.35 ± 0.04	2.21 ± 0.04	86.48	30.564^*^	< 0.001^*^
**t(p)**	2.47 (0.051)	7.19* (< 0.001)			

GM: Geometric mean Log_10_SD: Log_10_ Standard Deviation

t: Student t-test *: Statistically significant at *p* ≤ 0.001

Samples from the cultured colonies were taken to see whether they would have Gram-positive or Gram-negative bacteria (i.e., while some Gram-positive bacteria can cause illness, a significant portion of Gram-negative bacteria are particularly concerning. Many Gram-negative bacteria are pathogenic and can also show resistance to antibiotics, making them difficult to treat. Therefore, a key goal of enhanced cleaning protocols is to reduce the presence of these harmful Gram-negative bacteria. In our study, after implementing the enhanced cleaning procedures, finding fewer Gram-negative bacteria colonies in these cultures indicates the effectiveness of the cleaning and disinfection in reducing their numbers).

Table 4 indicates that the number of samples that detected the presence of Gram-negative bacteria isolated from high-touch areas was 95 samples (45.6%) before the enhanced cleaning, while it became 34 samples (16.3%) of the total samples after the enhanced cleaning with a statistically significant difference (*p* < 0.001). Also, Table 4 shows that the number of samples detected with Gram-negative bacteria isolated from high-touch areas was 76 (47.5%) before routine cleaning, while it was 64 (40%) after routine cleaning with no significant difference. The percentage of Gram-negative bacteria isolated from high-touch areas after enhanced cleaning was 16.3% of the total samples, while it was 40% after routine cleaning, with a statistically significant difference (*p* < 0.001).

**Table 4:** Gram-negative and Gram-positive isolates in samples taken from high touch areas in rooms A and B

	Type of bacteria	Before cleaning		After cleaning		χ^2^	*p*
		Number of isolates	% of total isolates	Number of isolates	% of total isolates		
Room A				After enhanced cleaning			
	Gram-negative	95	45.7	**34**	**16.3**	42.525^*^	< 0.001^*^
	Gram-positive	102	49	162	77.9		
	Mixed	11	5.3	12	5.8		
Room B				After routine cleaning			
	Gram-negative	76	47.5	**64**	**40**	1.831	0.400
	Gram-positive	68	42.5	78	48.8		
	Mixed	16	10	18	11.2		
Difference in Gram-negative samples between Room A and B after cleaning (**in bold**)						25.897^*^	< 0.001*

χ^2^: chi-squared test *: Statistically significant at *p* ≤ 0.001

Table 5 demonstrates the infections occurred in Room A and Room B six months before and after conducting the study. Based on the McNemar test results, the enhanced cleaning intervention in Room A appears to have had a significant impact on reducing total infections (*p* < 0.05). However, the lack of discordant pairs in Room B prevents a definitive conclusion about the effectiveness of the intervention in that room. As for ICU-acquired infection, the McNemar test showed non-significant results due to the small numbers. For more details see Table 3 in Supplementary File III.

**Table 5:** Infections in the neurosurgery ICU (Rooms A and B) 6 months before and 6 months after conducting the study

	Time	Room A	Room B	Test of significance
Total infection	6-months before intervention	18	15	χ^2^ = 0.87 *p* = 0.35
	6-months after intervention	11	15	
	Test of significance	McNemar**=** 5.14, *p* < 0.05	-	
ICU -acquired infections	6-months before intervention	6	4	χ^2^ = 1.07 *p* = 0.3
	6-months after intervention	2	4	
	Test of significance	McNemar**=** 2.25, *p* > 0.05	-	
Other infections	6-months before intervention	12	11	χ^2^ = 0.22 *p* = 0.64
	6-months after intervention	9	11	
	Test of significance	McNemar**=** 1.33, *p* > 0.05	-	

The McNemar test could not be performed in Room B due to a lack of changes in the number of infections between before and after the intervention. χ^2^: chi-squared test p: *p*-value

## Discussion

This study investigated the impact of enhanced cleaning protocols on bacterial counts within ICU high-touch surfaces. We began by assessing the ICU cleaning workers and nurses’ knowledge of proper cleaning and disinfection techniques. The initial assessment revealed a knowledge gap among the healthcare cleaning workers and nurses, with a median score and interquartile range (IQR) of 51 (40–60), as there were many areas of defects in their information. Previous studies highlight the crucial role of healthcare cleaning workers and nurses in preventing the spread of pathogens, especially in LMICs. When these workers lack proper cleaning knowledge, their hands, medical equipment, and the surfaces they touch can become contaminated with germs. This can inadvertently lead to the transmission of pathogens to patients, potentially causing infections. This problem is particularly evident in limited-resources healthcare facilities [2, 27–29].

Initial visual inspections suggested that a majority of surfaces were clean. However, subsequent environmental swabbing and culturing revealed elevated bacterial counts on these surfaces, indicating a discrepancy between visual cleanliness and actual bacterial contamination. This finding aligns with previous research by Huang et al. (2015) [25], which emphasized the limitations of relying solely on visual inspection to assess surface disinfection, particularly in critical hospital areas. Bacterial contamination can often be microscopic and invisible without proper testing methods, especially in LMICs where factors such as inadequate cleaning, work overload, high staff turnover, and limited resources contribute to increased contamination risks [2, 29, 30].

So, we implemented an educational program on enhanced cleaning to educate the healthcare cleaning workers and nurses about proper cleaning and disinfection (includes using dedicated supplies in intensive care units, employing proper cleaning techniques, ensuring adequate hand hygiene, following recommended cleaning frequencies, correctly preparing and using disinfectants, and effectively managing blood and body fluid spills) according to the CDC guidelines [22, 23], hoping to reduce the TACC on the environmental surfaces especially considering the low cost of implementing enhanced cleaning, which is a crucial factor in resource-limited hospitals and countries [29, 30]. The effect of this educational program appeared in the results of the samples taken from the high-touched areas (area around the beds, doorknobs, and nursing counters) after the enhanced cleaning. Bacterial counts on the environmental surfaces significantly decreased (*p* ≤ 0.001) after cleaning with the enhanced procedure, and the TACC became below the cut-off level (5 CFU/cm^2^) [12].

In the same vein, the NeoCLEAN study (2021) implemented a comprehensive strategy to enhance environmental cleaning in a resource-limited neonatal ICU. The intervention included training staff in proper cleaning methods, providing in-room cleaning wipes and checklists, and conducting regular cleaning audits with feedback. This multimodal approach led to significant improvements in cleaning practices and reduced bacterial contamination on surfaces and equipment [31]. Several studies have highlighted the need for a multi-factorial approach to overcome the challenges faced by healthcare staff in LMICs. These challenges include inadequate training in IPC, PPE shortages, inadequate disinfectants, insufficient hand hygiene, poor waste management, and work overload. One key strategy is to enhance proper cleaning and disinfection of environmental surfaces in hospitals. A multi-factorial approach involves developing evidence-based policies and processes, selecting appropriate cleaning and disinfecting products, educating staff, monitoring adherence to proper procedures, and implementing proper surveillance [28, 29, 31]. Most of these steps were applied by our study, and hence the significant reduction in TACC was obtained.

The current study reported a marked reduction in the percentage of Gram-negative isolates after the enhanced cleaning in room A. Compared to routine cleaning, the enhanced cleaning method showed a statistically significant difference in reducing Gram-negative bacteria on ICU surfaces. Following the enhanced cleaning, only 16.3% of the total bacterial colonies were Gram-negative, compared to 40% after routine cleaning. Henriksen et al. (2019) stated that Gram-negative bacteria outbreaks with antimicrobial resistance, especially those related to HAI, had an intimate relationship with the surrounding environment [32]. Studies by Huang et al. (2020) [11] and Gan et al. (2017) [33] in China support this connection. These studies found that enhanced cleaning and disinfection led to a decrease in multidrug-resistant organisms (MDRO) colonizing patients in the ICU.

Our study demonstrated a significant reduction in total infections in the ICU following the implementation of enhanced cleaning procedures. While ICU-acquired infections also decreased, the small sample size precluded a statistically significant finding. However, these results suggest that a larger-scale study could reveal a more substantial impact on ICU-acquired infections. This aligns with existing research highlighting the importance of improved hospital cleaning and disinfection practices. These practices help reduce the presence of pathogenic microorganisms on surfaces, which can lead to fewer HAI [34, 35]. Enhanced cleaning can play a crucial role in overcoming the challenges faced by healthcare facilities in LMICs. By implementing proper training and effective cleaning and disinfection practices, hospitals can mitigate the impact of factors such as limited resources, and insufficient infrastructure, hence, reducing the risk of HAI [22]. Through enhanced cleaning, hospitals can create a safer environment for patients and healthcare workers, leads to a shorter ICU stay and a significantly reduced morbidity and mortality rate [11].

It is evident from our study that the knowledge of the healthcare cleaning workers and nurses was suboptimal, and the need for regular training on proper cleaning and disinfection procedures is mandatory. By implementing comprehensive cleaning protocols as recommended by the CDC, the bacterial load, especially pathogenic species, could be reduced dramatically, and healthcare facilities can create a safer environment that reflects the significant decrease in the burden of HAI.

### Limitations and strength of the study

Our study has some limitations. Firstly, it was conducted in a relatively small ICU. To confirm the effectiveness of enhanced cleaning, we would need to repeat the study in larger healthcare settings. Secondly, the assessment of the healthcare cleaning workers and nurses was done through a questionnaire and an observational check list held before carrying out the educational program only. Hand hygiene assessed only before the intervention. While we did not conduct a formal post-intervention assessment the improvement of knowledge, compliance rates of hand hygiene and standard and transmission-based precautions, we indirectly evaluated its effectiveness by observing a significant reduction in bacterial load and HAI following the program. This reduction suggests that the knowledge and compliance rates likely improved, contributing to the positive outcomes. Thirdly, the Room A was receiving relatively more numbers of patients than room B. Despite these limitations, our study has several strengths. Firstly, the enhanced cleaning technique used is simple, affordable, and easy to implement, making it suitable for healthcare facilities with limited resources. Secondly, we evaluated the effectiveness of cleaning through various objective measures. This included measuring bacterial counts, identifying the types of bacteria present, and comparing HAI rates before and after the intervention. Third, we utilized a variety of educational methods to enhance learning, including audio-visual presentations, posters, videos, group discussions, and brainstorming sessions.

## Conclusions

From the current study, we can conclude that the knowledge of the healthcare cleaning workers and nurses was inadequate. While initial visual assessment suggested clean surfaces, bacterial cultures revealed significant levels of contamination before the intervention. Following the implementation of an educational program on enhanced cleaning methods aligned with CDC guidelines, a substantial reduction in TACC (below 5 CFU/cm²) was observed. Furthermore, the enhanced cleaning technique significantly reduced Gram-negative bacterial contamination. This decrease in contamination coincided with a decline in total infection rates documented in hospital records for Room A.

## Electronic supplementary material

Below is the link to the electronic supplementary material.

Supplementary Material 1

Supplementary Material 2

Supplementary Material 3

## Data Availability

The datasets used and/or analyzed during the current study are available from the corresponding author on reasonable request.

## Abbreviations

χ^2^: Chi-square test
ASA: The American Society of Anesthesiologists
CDC: Centers for Disease Control and Prevention
CFU: Colony Forming Unit
GM: Geometric mean
HAI: Healthcare acquired infection
ICU: Intensive care unit
IPC: Infection prevention and control
LMICs: Low-income and middle-income countries
Log_10_SD: Log_10_ Standard Deviation
MDRO: Multidrug-resistant organism
ppm: Parts per million
SPSS: The Statistical Package for Social Science
t: Student t-test
TACC: The Total Aerobic Colony Count
U: Mann Whitney test
