# Supplementary material for: The impact of enhanced cleaning on bacterial contamination of the hospital environmental surfaces: a clinical trial in critical care unit in an Egyptian hospital
Source: Antimicrob Resist Infect Control. 2024 Nov 19;13:138. doi: 10.1186/s13756-024-01489-z (PMC11575196; doi:10.1186/s13756-024-01489-z)
Supplement: Supplementary file 1 — Supplementary Material 1 [file 13756_2024_1489_MOESM1_ESM.docx]

**Supplementary File I**

**Interview questionnaire to assess the knowledge** **about cleaning technique**

**رقم الاستمارة........................**

**التاريخ........................**

**البيانات الشخصية**

1. **الاسم........................**
2. **السن ................**
3. **النوع**
   1. ذكر
   2. أنثي
4. **أعلي مؤهل دراسي حصلت عليه**
   1. الشهادة الابتدائية
   2. الشهادة الإعدادية
   3. الشهادة الثانوية
   4. دبلوم
   5. الشهادة الجامعية
5. **الوظيفة**
   1. ممرض
   2. عامل نظافة
6. **الحالة الإجتماعية**
   1. **أعزب**
   2. **متزوج**

نرجو التكرم باختيار ما يناسبك من اجابات على الأسئلة التالية بخصوص درجة المعرفة بالتنظيف والتطهير للاسطح البيئية بالمستشفي

1. **نظافة وتعقيم الايدي هو عامل مهم في الحد من العدوي**
   1. نعم
   2. لا
2. **يجب تنظيف الايدي قبل وبعد التعامل مع المريض وتنظيف الاسطح**
   1. نعم
   2. لا
3. **يعد ارتداء معدات الوقاية الشخصية المناسبة أمرًا مهمًا في الوقاية من العدوى المرتبطة بالرعاية الصحية**
   1. نعم
   2. لا
4. **ممارسات النظافة التنفسية مثل تغطية الفم والانف في حالة العطس او الكحة والحفاظ على مسافة آمنة لا تقلل من العدوى المرتبطة بالرعاية الصحية**
   1. نعم
   2. لا
5. **هل تم تدريبكم علي كيفية التنظيف؟**
6. نعم
7. لا
8. **هل يوجد اشراف عليكم أثناء التنظيف؟ (اختر إجابة واحدة)**
9. دائما
10. بعض الأحيان
11. أبدا
12. **كم مرة يجب تنظيف مناطق المريض؟ (اختر إجابة واحدة)**
13. يوميا
14. أسبوعياً
15. بعد خروج المريض
16. أبدا
17. **ما هى أهداف التنظيف اليومى فى المرافق الصحية؟ (اختر كل الاجابات الصحيحة)**
18. منع الانتشار المباشر من الكائينات الحية الدقيقة
19. توفير بيئة نظيفة ومريحة للمريض
20. تعزيز والحفاظ على عادات النظافة
21. **كيف يتم تنظيف الأماكن المختلفة بالمستشفى؟ (اختر كل الاجابات الصحيحة)**
22. البدء من أسفل لأعلى
23. البدء من أعلى لأسفل
24. البدء من الخارج للداخل
25. البدء من الداخل للخارج
26. **كيف يتم تنظيف أرضيات المستشفى؟ (اختر إجابة واحدة)**
27. كنس
28. التطهير باستخدام الماء
29. التطهير باستخدام الماء والصابون
30. التطهير باستخدام الماء والكلور
31. **ما هى المواد المستخدمة لتنظيف الأسطح داخل المستشفى؟ (اختر إجابة واحدة)**
32. قطعة قماش جافة
33. قطعة قماش مبللة بالماء فقط
34. قطعة قماش مبللة بالماء والصابون
35. قطعة قماش مبللة بالكلور
36. **ما ينبغى القيام به فى حال حدوث تسرب (الإفرازات/ التصريف، والدم، افرازات)؟ (اختر كل الاجابات الصحيحة)**
37. تمسح المنطقة بالماء
38. يتم تنظيف الانسكاب باستخدام منشفة ورقية ومطهر
39. يتم جمع الانسكاب، ووضعه فى كيس من البلاستيك، ثم وصفه بأنه "النفايات الصلبة الملوثة"
40. يجب استخدام قفازات
41. يجب مسح المنطقة باستخدام محلول الكلور
42. **ما هى الأدوات المستخدمة اللازمة للتنظيف (اختر كل الاجابات الصحيحة)**
43. جردل واحد
44. اثنين من الجرادل – واحدة مع المياه النظيفة. والآخر مع الماء والصابون.
45. قطعة قماش
46. قفازات غير معقمة
47. قفازات معقمة
48. قفازات مطاطية
49. ممسحة
50. **ما هى السوائل التى تستخدم للتنظيف فى المستشفى؟ (اختر كل الاجابات الصحيحة)**
51. لا يوجد
52. الماء والصابون
53. الكلور
54. الكلورهيكسيدين جلوكونات
55. **ما هى السوائل التى تستخدم للتطهير فى المستشفى؟ (اختر كل الاجابات الصحيحة)**
56. لا يوجد
57. الماء والصابون
58. الكلور
59. الكلورهيكسيدين جلوكونات
60. الديتول
61. **أين تغسل المساحات والقماش؟**
62. فى الأحواض المخصصة للمرضى
63. فى أحواض مصممة خارج منطقة المرضى
64. **متى يجب غسل أدوات التنظيف (اختر كل الاجابات الصحيحة)**
65. بعد كل إجراء تنظيف
66. يوميا
67. اسبوعيا
68. **ما يحدث لأدوات التنظيف بعد التنظيف؟ (اختر إجابة واحدة)**
69. يتم استخدامه على الفور لتنظيف أى مكان آخر
70. يتم غسلها وتخزينها عندما تكون مبتلة
71. يتم غسلها وتركها لتجف
