# Supplementary material for: The impact of enhanced cleaning on bacterial contamination of the hospital environmental surfaces: a clinical trial in critical care unit in an Egyptian hospital
Source: Antimicrob Resist Infect Control. 2024 Nov 19;13:138. doi: 10.1186/s13756-024-01489-z (PMC11575196; doi:10.1186/s13756-024-01489-z)
Supplement: Supplementary file 2 — Supplementary Material 2 [file 13756_2024_1489_MOESM2_ESM.docx]

**Supplementary File II**

**
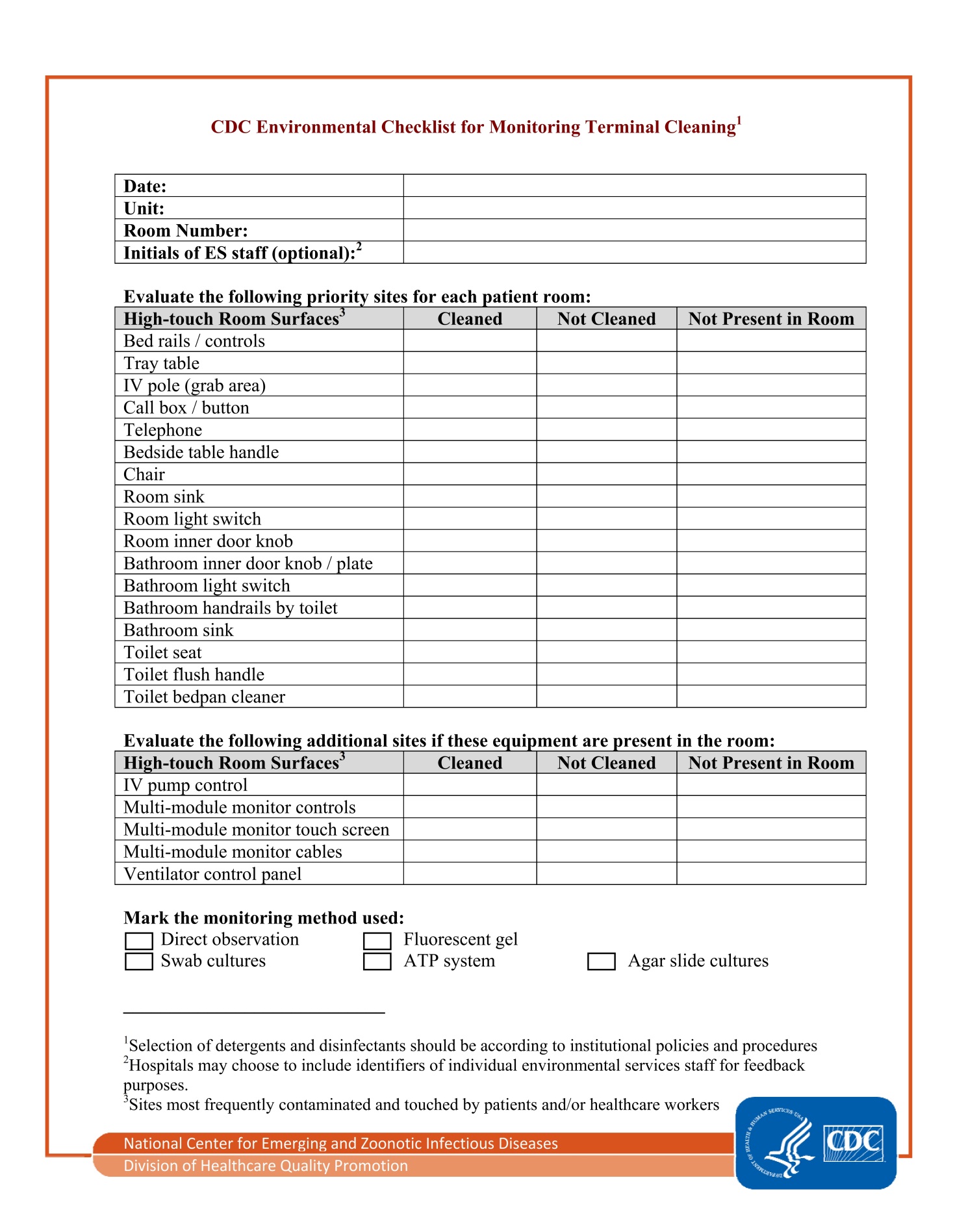
**

<https://www.cdc.gov/hai/pdfs/toolkits/environmental-cleaning-checklist-10-6-2010.pdf>
