# Supplementary material for: The impact of enhanced cleaning on bacterial contamination of the hospital environmental surfaces: a clinical trial in critical care unit in an Egyptian hospital
Source: Antimicrob Resist Infect Control. 2024 Nov 19;13:138. doi: 10.1186/s13756-024-01489-z (PMC11575196; doi:10.1186/s13756-024-01489-z)
Supplement: Supplementary file 3 — Supplementary Material 3 [file 13756_2024_1489_MOESM3_ESM.docx]

**Supplementary File III**

Table 1: Distribution of the socio-demographic characteristics of the participants

|  | **Room A** | **Room B** | **Test of significance (p)** |
| --- | --- | --- | --- |
| **Workers** | **n = 10 (%)** | **n = 10 (%)** |  |
| Age  18 –  31 +  Mean (years) | 3 (30.0%)  7 (70.0%)  37 | 2 (20.0%)  8 (80.0%)  38.5 | **χ^2^**=0.267, p=0.61  t= -0.32, p=0.38 |
| Sex   - Males - Females | 4 (40.0%)  6 (60.0%) | 6 (60.0%)  4 (40.0%) | **χ^2^**=0.8, p=0.37 |
| Educational level completed   - Primary education - Secondary education - University education | 8 (80.0%)  1 (10.0%)  1 (10.0%) | 6 (60.0%)  3 (30.0%)  1 (10.0%) | **χ^2^**=1.29, p=0.53 |
| Marital status   - Married | 10 (100.0%) | 10 (100.0%) | **χ^2^**=0.0, p=1 |
| **Nurses** | **n = 8 (%)** | **n = 8 (%)** |  |
| Age  18 –  31 +  Mean (years) | 3 (37.5%)  5 (62.5%)  33.5 | 2 (25.0%)  6 (75.0%)  36.5 | **χ^2^**=0.29, p=0.59  t=0.97, p=0.17 |
| Sex   - Females | 8 (100.0%) | 8 (100.0%) | **χ^2^**=0.0, p=1 |
| Educational level completed   - Secondary education - Diploma education - University education | 6 (75.0%)  1 (12.5%)  1 (12.5%) | 6 (75.0%)  1 (12.5%)  1 (12.5%) | **χ^2^**=0.0, p=1 |
| Marital status   - Married | 8 (100.0%) | 8 (100.0%) | **χ^2^**=0.0, p=1 |

**χ^2^: chi square test t: t-test p: p-value**

Table (2): Assessment of the knowledge of the cleaning team in the ICU of the neurosurgery department

| **Topics of questions** | **Answers** | **n=36** | **%** |
| --- | --- | --- | --- |
| Proper hand hygiene is important factor in reducing HAI | - Yes ^#^ - No | 10  26 | 27.8%  72.2% |
| Proper hand washing should be applied before and after dealing with patients | - Yes ^#^ - No | 18  18 | 50.0%  50.0% |
| Proper hand washing should be applied before and after cleaning the environmental surfaces | - Yes ^#^ - No | 16  20 | 44.4%  55.6% |
| Wearing appropriate personal protective equipment is important in preventing HAI | - Yes ^#^ - No | 25  11 | 69.4%  30.6% |
| Respiratory hygiene practices and maintaining a safe distance do not reduce the HAI | - Yes - No ^#^ | 10  26 | 27.8%  72.2% |
| Previous training about the techniques of cleaning | - Yes ^#^ - No | 20  16 | 55.6%  44.4% |
| Supervision while cleaning | - Yes ^#^ - No | 24  12 | 66.7%  33.3% |
| Frequency of cleaning per day | - Once - Other | 36  0 | 100%  0% |
| Goal of daily cleaning in the hospital | - Prevent direct spread to microorganisms. ^#^ - Provide a clean and comfortable environment to the patient. | 6  30 | 16.7%  83.3% |
| Directions of cleaning the ICU rooms | - Complete correct answer - Incomplete correct answer - Wrong answer | 8  20  8 | 22.2%  55.6%  22.2% |
| The proper techniques of cleaning in ICU | - Correct answer (disinfection by sodium hypochlorite) - Wrong answer | 18  18 | 50.0%  50.0% |
| Materials used to clean the surfaces inside the ICU rooms | - Pieces of cloth dampened with water and chlorine.^#^ - Pieces of cloth dampened with water and soap. | 24  12 | 66.7%  33.3% |
| Proper procedure in case of spills (human exudates or blood) | - Complete correct answer - Incomplete correct answer - Wrong answer | 4  22  10 | 11.1%  61.1%  27.8% |
| Tools used during cleaning | - Complete correct answer (Two buckets, mop, rubber gloves) - Incomplete correct answer - Wrong answer | 4  20  12 | 11.1%  55.6%  33.3% |
| Liquids used during cleaning the surfaces of the ICU | - Soap and water. ^#^ - Chlorine. | 12  24 | 33.3%  66.7% |
| Liquids used during disinfection the surfaces of the ICU | - Soap and water. - Chlorine. ^#^ | 18  18 | 50.0%  50.0% |
| Place of the sinks used to wash wipers and cloths for cleaning | - Outside patients areas ^#^ - Inside patients areas | 36  0 | 100.0%  0.0% |
| Frequency of washing cleaning tools | - After each cleaning procedure ^#^ - Daily | 12  24 | 33.3%  66.7% |
| Time to use cleaning tools after washing | - Leave them to dry first. ^#^ - Use them immediately without drying. | 18  18 | 50.0%  50.0% |

* Not mutually exclusive **^#^** Correct answer

Table (3): Infections in the neurosurgery ICU 6 months before and 6 months after conducting the study

|  | **6-months Before the intervention** | | | | | | **6-months After the intervention** | | | | | |
| --- | --- | --- | --- | --- | --- | --- | --- | --- | --- | --- | --- | --- |
|  | **Total infections recorded** | | **ICU -acquired infections** | | **Other infections** | | **Total infections** | | **ICU -acquired infections** | | **Other infections** | |
|  | **No.** | **%** | **No.** | **%** | **No.** | **%** | **No.** | **%** | **No.** | **%** | **N0.** | **%** |
| ICU (Both rooms) | 33 | 100 | 10 | 30.0 | 23 | 70.0 | 26 | 100 | 6 | 23.1 | 20 | 76.9 |
| Room A | 18 | 100 | 6 | 33.3 | 12 | 66.7 | 11 | 100 | 2 | 18.2 | 9 | 81.8 |
| Room B | 15 | 100 | 4 | 26.7 | 11 | 73.3 | 15 | 100 | 4 | 26.7 | 11 | 73.3 |
